# New insights into the function of a versatile class of membrane molecular motors from studies of *Myxococcus xanthus* surface (gliding) motility

**DOI:** 10.15698/mic2017.03.563

**Published:** 2017-03-02

**Authors:** Tâm Mignot, Marcelo Nöllmann

**Affiliations:** 1 Laboratoire de Chimie Bactérienne, Institut de Microbiologie de la Méditerranée, CNRS -Aix Marseille University UMR7283, 31 chemin Joseph Aiguier, 13009 Marseille, France.; 2 Centre de Biochimie Structurale, CNRS UMR5048, INSERM U1054, Montpellier University, 29 rue de Navacelles, 34090 Montpellier, France.

**Keywords:** molecular motors, motility, bacterial cell envelope, proton channel, adhesion

## Abstract

Cell motility is a central function of living cells, as it empowers colonization of new environmental niches, cooperation, and development of multicellular organisms. This process is achieved by complex yet precise energy-consuming machineries in both eukaryotes and bacteria. Bacteria move on surfaces using extracellular appendages such as flagella and pili but also by a less-understood process called gliding motility. During this process, rod-shaped bacteria move smoothly along their long axis without any visible morphological changes besides occasional bending. For this reason, the molecular mechanism of gliding motility and its origin have long remained a complete mystery. An important breakthrough in the understanding of gliding motility came from single cell and genetic studies in the delta-proteobacterium *Myxococcus xanthus*. These early studies revealed, for the first time, the existence of bacterial Focal Adhesion complexes (FA). FAs are formed at the bacterial pole and rapidly move towards the opposite cell pole. Their attachment to the underlying surface is linked to cell propulsion, in a process similar to the rearward translocation of actomyosin complexes in Apicomplexans. The protein machinery that forms at FAs was shown to contain up to seventeen proteins predicted to localize in all layers of the bacterial cell envelope, the cytosolic face, the inner membrane (IM), the periplasmic space and the outer membrane (OM). Among these proteins, a proton-gated channel at the inner membrane was identified as the molecular motor. Thus, thrust generation requires the transduction of traction forces generated at the inner membrane through the cell envelope beyond the rigid barrier of the bacterial peptidoglycan.

In a recent study, we combined microfluidics and Total Internal Reflection Microscopy (TIRFM) to follow the dynamics of motility proteins at FAs during motility at high temporal resolution (**Faure, *et al.* Nature 2016**). We found that the gliding machinery is formed by three major protein subcomplexes: (1) an inner membrane platform assembled on a scaffold formed by the bacterial actin cytoskeleton, (2) a proton-motive-force-energized molecular motor, and (3) a periplasmic-OM complex. Strikingly, we found that dynamic FA complexes leaving the leading pole become propulsive only upon surface immobilization (Figure 1A). During their dynamic phase, these complexes follow a helical right-handed helical path (Figure 1A). Upon surface attachment, this right-handed helical movement leads to a left-handed rotation of the cell around its long axis (Figure 1A). But what is the molecular basis of this process?

**Figure 1:**
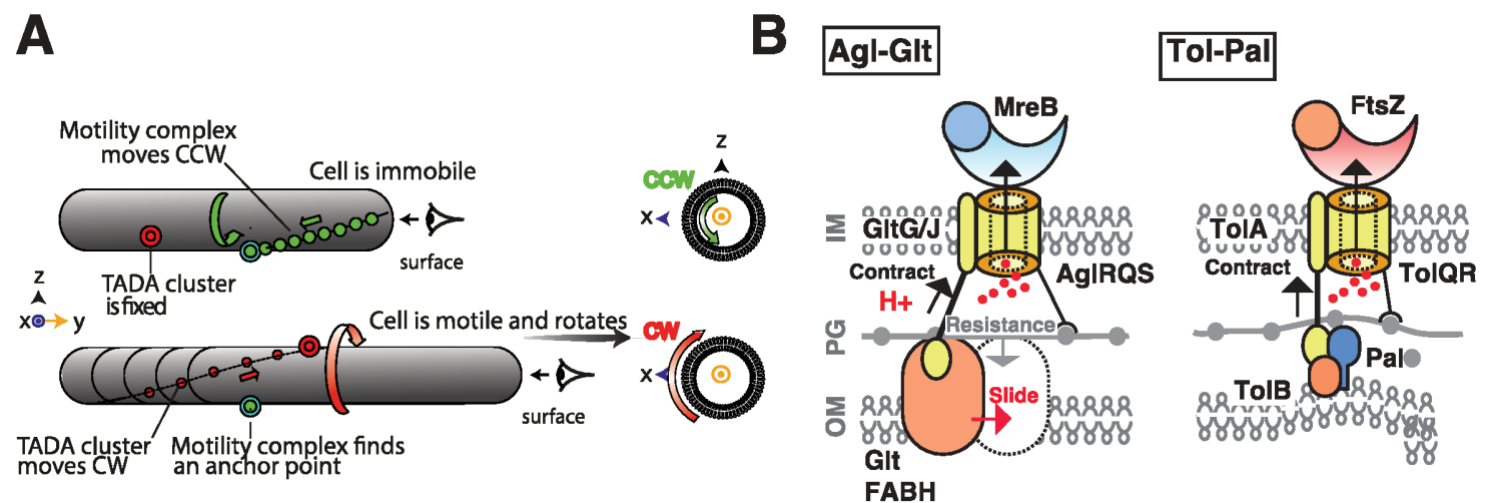
FIGURE 1 **(A) **The helical movement of intracellular motors drives rotational movement of the cell. Intracellular counter-clockwise (CCW)- motor complexes (green) propel the cell in a screw-like clockwise direction when they become tethered at so-called FAs. (CW, shown by trajectory of a fixed point at the cell surface in red. The CCW or CW directionality is determined by the position of the observer (eye symbol). *Adapted from Faure et al, 2016 with permission.* **(B)** Core structure and function of the Tol/Exb/Agl class of molecular motors. The Agl-Glt and Tol-Pal systems are shown side-by-side for comparison. The structure of the Agl-Glt machinery is simplified to its core Tol-like components for clarity. In both cases, the proton flow through a PG-bound TolQR-type channel (yellow) is believed to energize cyclic interactions between a flexible IM-anchored periplasmic TonBC domain protein (black-yellow) and TonB-box proteins in the OM (orange). In the case of Agl-Glt, combined with the rigid anchoring to PG (grey arrow), this activity would push the OM protein laterally (red arrow), propelling the cell. In Tol-Pal, similar dynamics would provoke OM invagination and participate in cytokinesis. Interestingly, both Tol-Pal and Agl-Glt are physically connected to cytoskeletal proteins, FtsZ and MreB respectively. *Adapted from Faure et al, 2016 with permission.*

To address this question, we followed the *in vivo* dynamics of pairs of proteins belonging to different sub-complexes of the gliding apparatus. Uniquely, we found that the motor processively powers the movement of cytosolic-IM subcomplexes from the leading pole to the adhesion site, where interactions with periplasm-OM subcomplexes can take place. In fact, periplasm-OM proteins are distributed homogeneously around the cell envelope but become actively recruited by the mobile IM complex at FAs. These dynamic interactions tether the machinery to the substrate because the OM complex is strongly adhesive under tension. In fact, adhesions are so strong that the only way cells can continue moving forward is by leaving these adhesive parts behind! We envision that these may constitute part of the trail left by gliding cells serving as a pheromone to allow other *Myxococcus *cells to follow the steps of their siblings.

This model, however, poses an intriguing question: how do IM and OM subcomplexes transiently interact through the dense and rigid peptidoglycan (PC) meshwork? Recently, we revealed by fine-grained bioinformatics the predictive structure of the motility complex. Strikingly, some components of the gliding apparatus bear a resemblance with proteins of Tol/Exb systems. In Gram negative bacteria, these molecular complexes are respectively involved in cell division and macromolecule import such as iron siderophores. How they exactly operate is not clear but both the Tol and Exb systems assemble a proton-conducting channel in the bacterial inner membrane like the Agl system. In the Tol and Exb systems, the IM channel (formed by TolQR and ExbBD) also interacts dynamically with OM proteins through PG in a pmf-dependent way. How this interaction exactly occurs in not completely clear but in both cases it involves a channel-interacting IM protein (TolA or TonB in the Tol and Exb systems, respectively) with an extended periplasmic coiled-coil domain and a conserved globular Ct-domain called TonBC. A large body of biochemical evidence suggests that a pmf-driven conformational change of the coiled-coil unfolds the protein from the IM to the OM, reaching OM interacting proteins through the PG meshwork. However, while these contacts are essential for function, how they mediate function is unclear both in the Tol and Exb systems. But, do these similarities also attain the motor complex responsible for energizing the system?

The molecular motor at the core of the Agl-Glt apparatus, the AglRQS system, is phylogenetically related to the TolQR and ExbBD channels and contains conserved residues that are also critical for function (for example a conserved Asp that binds protons to the suspected lumen of the channel). Second, GltG and GltJ are modular TolA/TonB-like proteins, specifically sharing a single transmembrane helix, a periplasmic helical domain, and TonBC motifs. GltG directly interacts with AglR, the TolQ homolog, suggesting that it could also provoke pmf-dependent contacts with the OM. In the OM, the predicted β-barrel proteins GltAB contain possible TonB boxes and could thus provide contact sites for GltG. Thus, the Agl-Glt machinery is equipped with domains that trigger dynamic interactions between the OM and the IM despite the presence of PG. However, the TolQR systems are involved in contractile motions. Is this type of movement also a key for the Agl-Glt system?

In fact, Agl-driven contractions became apparent in *Myxococcus *cells, in which sporulation was artificially induced. In these cells, sporulation is induced by the addition of Glycerol which provokes the rapid remodeling of PG. Remarkably, a fraction of cells entering sporulation were still motile and formed conspicuous constrictions that, similar to FA complexes, remained fixed relative to the surface. These constrictions likely result from the activity of the Agl-Glt machinery because the Agl-Glt complex localized preferentially to the constriction site. To explain these results we proposed that pmf-driven cyclic interactions between the IM platform and the OM adhesion complex drive the directed movements of the system across the multilayered cell envelope (Figure 1B). In sporulating cells, these dynamic Agl-Glt connections may be unmasked due to the weakened PG, revealing the contractile activity of the system.

Agl-Glt-type machineries are quite diverse and have been shown to mediate processes other than motility. For example, in *Myxococcus *the Nfs complex, a Glt-like apparatus, also associates with AglRQS but in this process, the Agl complex drives directed movements of Nfs proteins bound to the major spore coat polymer, thus spreading the polymer around the spore membrane. These motions could also be driven by cyclic contacts between the IM and the OM, which may be a common operating process of the emerging class of Tol/Exb/Agl systems (Figure 1B). In fact, a contractile activity of Tol-Pal is proposed to contribute to OM invagination during the final step of cell division (Figure 1B). Interestingly, other PMF-driven motors (i.e. the flagellar motor, the ATP synthase and the *Bacteroidetes* motility apparatus) are rotary devices. Thus, even though core pmf-utilizing components with a common evolutionary origin are shared by the Tol/Exb/Agl and flagellar motor systems, their coupling with accessory components has enabled very different mechanical outputs. This versatility could explain the lack of a universal bacterial gliding machinery. In fact, gliding machineries could have evolved several times from the specialization of a pmf-driven motor. Thus, given that Tol/Exb/Agl motor types are near ubiquitous in proteobacteria, we envision that they were widely recruited and accessorized independently, for multiple cell envelope processes, such as motility, transport, envelope biogenesis and cell division.

